# Renormalized self-intersection local time of bifractional Brownian motion

**DOI:** 10.1186/s13660-018-1916-3

**Published:** 2018-11-23

**Authors:** Zhenlong Chen, Liheng Sang, Xiaozhen Hao

**Affiliations:** 1 School of Statistics and Mathematics, Zhejiang Gongshang University, Hangzhou, China; 2 School of Mathematics and Finance, Chuzhou University, Chuzhou, China

**Keywords:** 60F05, 60G15, 60G18, Bifractional Brownian motion, Self-intersection local time, Renormalization, Existence

## Abstract

Let $B^{H,K}=\{B^{H,K}(t), t \geq 0\}$ be a *d*-dimensional bifractional Brownian motion with Hurst parameters $H\in (0,1)$ and $K\in (0,1]$. Assuming $d\geq 2$, we prove that the renormalized self-intersection local time

$$\begin{aligned} \int^{T}_{0} \int^{t}_{0}\delta \bigl(B^{H,K}(t)-B^{H,K}(s) \bigr)\,ds\,dt-\mathbb{E} \biggl( \int^{T}_{0} \int^{t}_{0}\delta \bigl(B^{H,K}(t)-B^{H,K}(s) \bigr)\,ds\,dt \biggr) \end{aligned}$$

exists in $L^{2}$ if and only if $HKd< 3/2$, where *δ* denotes the Dirac delta function. Our work generalizes the result of the renormalized self-intersection local time for fractional Brownian motion.

## Introduction

Fractional Brownian motion has received much attention in recent years due to its long-range dependence, stationarity increments, and self-similarity. It has been widely applied in turbulence, image processing, mathematics finance, and so on for small increments. However, it is inadequate to large increments. So, it is very natural to explore the extension of fractional Brownian motion which keeps some properties of fractional Brownian motion (gaussianity, stationarity of small increments, self-similarity), and then bifractional Brownian motion as a generalization of fractional Brownian motion has been investigated by many authors, see [5, 13, 15] and the references therein for more details.

Let us briefly recall some related definitions of bifractional Brownian motion as follows. Set $B^{H,K}_{0}=\{B^{H,K}_{0}(t), t \geq 0 \}$ be a bifractional Brownian motion in $\mathbb{R}$ with Hurst parameters $H\in (0,1)$ and $K\in (0,1]$, i.e., a centered, real-valued Gaussian process with zero mean and covariance function given by

1.1 $$\begin{aligned} \mathbb{E} \bigl[B^{H,K}_{0}(s)B^{H,K}_{0}(t) \bigr]=\frac{1}{2^{K}} \bigl[ \bigl(t^{2H}+s ^{2H} \bigr)^{K}-\vert t-s \vert ^{2HK} \bigr], \quad s,t \geq 0. \end{aligned}$$

This process is *HK*-self similar and satisfies the following estimates:

1.2 $$\begin{aligned} 2^{-K}\vert t-s \vert ^{2HK}\leq \mathbb{E} \bigl[ \bigl(B^{H,K}_{0}(t)-B^{H,K}_{0}(s) \bigr)^{2} \bigr] \leq 2^{1-K}\vert t-s \vert ^{2HK} \end{aligned}$$

for each $T>0$ and $s,t\in [0,T]$. Moreover, we can easily check that it is Hölder continuous of order *δ* for any $\delta < HK$ from the Kolmogorov criterium. In particular, if $K=1$, $B^{H,1}_{0}(t)$ is a fractional Brownian motion with Hurst parameter $H\in (0,1)$.

We associate with $B^{H,K}_{0}$ a Gaussian process $B^{H,K}=\{B^{H,K}(t), t \geq 0\}$ in $\mathbb{R}^{d}$ by

1.3 $$\begin{aligned} B^{H,K}(t)= \bigl(B^{H,K}_{1}(t),\ldots ,B^{H,K}_{d}(t) \bigr), \end{aligned}$$

where $B^{H,K}_{1},\ldots ,B^{H,K}_{d}$ are independent copies of $B^{H,K}_{0}$. $B^{H,K}$ is called a *d*-dimensional bifractional Brownian motion with Hurst parameters $H\in (0,1)$ and $K\in (0,1]$.

On the other hand, since the work of Varadhan [16], self-intersection local time, as an important topic of probability theory, has been widely considered and studied in recent years. Especially, when it comes to Brownian motion and fractional Brownian motion, it has been extensively studied, see [1, 2, 4, 6, 10, 11, 17] and the references therein.

Recently, the self-intersection local time of bifractional Brownian motion has already been researched by few scholars. Jiang and Wang [9] studied the existence and smoothness of the self-intersection local time of bifractional Brownian motions. Chen et al. [3] considered the existence and smoothness of self-intersection local times for a large class of Gaussian random fields, including fractional Brownian motion, fractional Brownian sheets, and bifractional Brownian motion. For more on the local time of bifractional Brownian motion, we can see [14, 18] and so on.

We know that the non-renormalized self-intersection local time of fractional Brownian motion exists in $L^{2}$ for $Hd<1$ by the results of Jiang and Wang [9] and Chen et al. [3]. But for the case of renormalization, Hu and Nualart [7] obtained that the renormalized self-intersection local time of fractional Brownian motion exists in $L^{2}$ for $Hd<3/2$. Therefore, the existence is different between renormalization and non-renormalization of self-intersection local time. In this paper, we consider the existence of renormalized self-intersection local time for bifractional Brownian motion. Our conclusions generalize the result of fractional Brownian motion in Hu and Nualart [7] to bifractional Brownian motions.

In this paper, the following local times of bifractional Brownian motion will be involved, including the local time $\ell_{T} ^{H,K}(x)$ and the self-intersection local time $I(H,K,T)$ of bifractional Brownian motion $B^{H,K}(t)$. Formally, they are defined respectively as follows: for $T>0$,

1.4 $$\begin{aligned} \ell_{T}^{H,K}(x)= \int^{T}_{0}{\delta } \bigl(B^{H,K}(t)-x \bigr)\,dt \end{aligned}$$

and

1.5 $$\begin{aligned} L(H,K,T)= \int^{T}_{0} \int^{t}_{0}\delta \bigl(B^{H,K}(t)-B^{H,K}(s) \bigr)\,ds\,dt, \end{aligned}$$

where ${\delta }(x)$ is the Dirac delta function for $x \in \mathbb{R}^{d}$.

The Dirac delta function is formally

1.6 $$\begin{aligned} \delta (x)=\lim_{\varepsilon \rightarrow 0}p_{\varepsilon }(x)=(2 \pi )^{-d} \int_{\mathbb{R}^{d}}\exp \bigl\{ i\langle\xi ,x\rangle \bigr\} \,d{\xi }, \end{aligned}$$

where

1.7 $$\begin{aligned} p_{\varepsilon }(x)=(2\pi \varepsilon )^{-\frac{d}{2}}\exp \biggl\{ -\frac{\vert x \vert ^{2}}{2 \varepsilon } \biggr\} =(2\pi )^{-d} \int_{\mathbb{R}^{d}}\exp \biggl\{ i\langle \xi ,x\rangle-\frac{1}{2} \varepsilon { \vert \xi \vert }^{2} \biggr\} \,d{\xi }. \end{aligned}$$

By (1.6), we define the approximated self-intersection local time of bifractional Brownian motion by

1.8 $$\begin{aligned} L_{\varepsilon }(H,K,T)= \int^{T}_{0} \int^{t}_{0}p_{\varepsilon } \bigl(B ^{H,K}(t)-B^{H,K}(s) \bigr)\,ds\,dt. \end{aligned}$$

We will consider the following two questions:

1. We consider the existence in $L^{2}$ and a sharp upper bound of second moment of local time $\ell_{T}^{H,K}(x)$ for bifractional Brownian motion. Although the existence of the local time for anisotropic Gaussian random fields is obtained in Theorem 2.6 by Chen et al. [3], which contains the result of bifractional Brownian motion, a sharp upper bound of second moment of the local time for anisotropic Gaussian random fields is not got. It is not enough to research the local time for Gaussian random fields. So, in order to fill this vacancy for bifractional Brownian motion, we give the following Theorem 1.1.

### Theorem 1.1

*Assume that* $HKd<1$. *Then the local time* $\ell_{T}^{H,K}(x)$ *of the* *d*-*dimension bifractional Brownian motion is square integrable*, *and for any* $x \in \mathbb{R}^{d}$, *we have the sharp upper bound*

$$ \mathbb{E} \bigl[\ell_{T}^{H,K}(x) \bigr]^{2}\leq \frac{2\varGamma^{2}(1-HKd)}{(2 \pi )^{d}k^{\frac{2}{d}}\varGamma (3-HKd)}T^{2-2HKd}, $$

*where* *k* *is a constant depending on* *H* *and K*.

(2) The latter problem is to generalize the result of Hu and Nualart [7] to bifractional Brownian motion. That is, we will consider the existence of the renormalized self-intersection local time of bifractional Brownian motion in $L^{2}$. We get the following Theorem 1.2.

### Theorem 1.2

*Let* $B^{H,K}=\{B^{H,K}(t), t \geq 0\}$ *be a* *d*-*dimensional bifractional Brownian motion with Hurst parameters* $H\in (0,1)$ *and* $K\in (0,1]$. *Then*, *for every* $T>0$, *the renormalized self*-*intersection local time* $L_{\varepsilon }(H,K,T)-\mathbb{E}[L _{\varepsilon }(H,K,T)]$ *of* $B^{H,K}$ *converges in* $L^{2}$ *as* *ε* *tends to zero if and only if* $HKd<\frac{3}{2}$.

The paper is organized as follows. In Sect. 2, we study the square-integrable of the local time of *d*-dimensional bifractional Brownian motion. We prove the existence of the self-intersection local time of *d*-dimensional bifractional Brownian motion in Sect. 3.

For simplicity, we will use *k* to denote unspecified positive finite constants which may be different in each appearance throughout this paper.

## Square integrable of the local time

In this section, the local time of the *d*-dimension bifractional Brownian motion will be discussed. We firstly give the following lemma which plays an important role in proving the existence of the local time and Theorem 1.1.

### Lemma 2.1

([15], Proposition 2.1)

*For all constants* $0< a< b$, $B^{H,K}_{0}(t)$ *is strongly locally* *φ*-*nondeterministic on* $I=[a,b]$ *with* $\varphi (r)=r^{2HK}$. *That is*, *there exist positive constants* *k* *and* $r_{0}$ *such that*, *for all* $t\in I$ *and all* $0< r\leq \min \{t, r_{0}\}$,

2.1 $$\begin{aligned} \operatorname{Var} \bigl(B^{H,K}_{0}(t)\mid B^{H,K}_{0}(s):s\in I, r \leq \vert s-t\vert \leq r_{0} \bigr)\geq k\varphi (r). \end{aligned}$$

*By* (2.1), *we have that*: *if* $0\leq t_{1}< t_{2}<\cdots <t _{n}<T$, *then there is a constant* $k>0$ *such that*

2.2 $$\begin{aligned} \operatorname{Var} \Biggl(\sum^{n}_{m=2}u_{m} \bigl(B^{H,K}_{0}(t_{m})-B^{H,K}_{0}(t _{m-1}) \bigr) \Biggr)\geq k\sum^{n}_{m=2}u_{m}^{2} \vert t_{m}-t_{m-1} \vert ^{2HK} \end{aligned}$$

*for any* $u_{m}\in \mathbb{R}$, $m=2,\ldots ,n$.

Now, we prove Theorem 1.1, which is an extension of Theorem 5.1 in [8] to bifractional Brownian motion.

### Proof of Theorem 1.1

From the expression of $\ell_{T}^{H,K}(x)$ and (1.6), we can get

2.3 $$\begin{aligned} &\mathbb{E} \bigl[ \ell_{T}^{H,K}(x) \bigr]^{2} \\ &\quad =\mathbb{E} \biggl[ \int_{[0,T]^{2}}\delta \bigl(B^{H,K}(t)-x \bigr)\delta \bigl(B^{H,K}(s)-x \bigr)\,ds\,dt \biggr] \\ &\quad =\frac{1}{(2\pi )^{2d}} \int_{[0,T]^{2}} \int_{\mathbb{R}^{2d}} \mathbb{E} \bigl[\exp \bigl\{ i \bigl[ \bigl\langle \bigl(B^{H,K}(t)-x \bigr),\xi \bigr\rangle + \bigl\langle \bigl(B^{H,K}(s)-x \bigr), \eta \bigr\rangle \bigr] \bigr\} \bigr]\,d\xi \,d\eta \,ds\,dt \\ &\quad =\frac{1}{(2\pi )^{2d}} \int_{[0,T]^{2}} \int_{\mathbb{R}^{2d}} \mathbb{E} \Biggl[\exp \Biggl\{ i\sum ^{d}_{m=1} \bigl[ \bigl(B^{H,K}_{m}(t) -x _{m} \bigr)\xi_{m} \\ &\quad \quad {}+ \bigl(B^{H,K}_{m}(s)-x_{m} \bigr) \eta_{m} \bigr] \Biggr\} \Biggr]\,d\xi\,d\eta \,ds\,dt \\ &\quad \leq \frac{2}{(2\pi )^{2d}} \int_{0\leq s< t\leq T} \int_{\mathbb{R} ^{2d}} \prod^{d}_{m=1} \exp \biggl\{ -\frac{1}{2}\operatorname{Var}( \bigl[ \bigl(B^{H,K}_{m}(t) \xi_{m} \\ &\quad \quad {}+B^{H,K}_{m}(s)\eta_{m} \bigr] \bigr) \biggr\} \,d\xi \,d\eta \,ds\,dt, \end{aligned}$$

where we used the fact that $\mathbb{E}[e^{iX}]=\exp \{-\frac{1}{2} \operatorname{Var}(X)\}$ for any Gaussian random variable *X*.

By the local nondeterminism (2.2) of bifractional Brownian motion and $\operatorname{Var}(B^{H,K}(t))=t^{2HK}$, we have that, for $0\leq s< t \leq T$, there is a positive constant $k>0$ such that

$$\begin{aligned} \operatorname{Var} \bigl(B^{H,K}_{m}(t)\xi_{m}+B^{H,K}_{m}(s) \eta_{m} \bigr) &= \operatorname{Var} \bigl( \bigl(B^{H,K}_{m}(t)-B^{H,K}_{m}(s) \bigr)\xi_{m} \bigr)+\operatorname{Var} \bigl(B^{H,K}_{m}(s) (\xi_{m}+\eta_{m}) \bigr) \\ &\geq k \bigl[{\vert \xi_{m} \vert }^{2}\vert t-s \vert ^{2HK}+\vert \xi_{m}+\eta_{m} \vert ^{2}s^{2HK} \bigr]. \end{aligned}$$

Therefore, we get that the last integral of (2.3) is bounded by the following expression:

2.4 $$\begin{aligned} \frac{2}{(2\pi )^{2d}} \int_{0\leq s< t\leq T} \int_{{\mathbb{R}}^{2d}} \prod^{d}_{m=1} \exp \biggl\{ -\frac{k}{2} \bigl[{\vert \xi_{m} \vert }^{2}\vert t-s \vert ^{2HK}+\vert \xi_{m}+ \eta_{m} \vert ^{2}s^{2HK} \bigr] \biggr\} \,d\xi \,d\eta \,dt\,ds. \end{aligned}$$

By integrating with respect to *ξ* and *η*, respectively, and changing the variable $s=tu$ for *s*, we obtain that expression (2.4) is equal to

2.5 $$\begin{aligned} &\frac{2}{(2\pi )^{d}k^{\frac{d}{2}}} \int_{0\leq s< t\leq T}\frac{1}{s ^{HKd}\vert t-s \vert ^{HKd}}\,ds\,dt \\ &\quad =\frac{2}{(2\pi )^{d}k^{\frac{d}{2}}} \int_{0}^{T}\,dt \int_{0}^{t}s ^{-HKd} (t-s)^{-HKd} \,ds \\ &\quad =\frac{2}{(2\pi )^{d}k^{\frac{d}{2}}} \int_{0}^{T}t^{(1-2HKd)}\,dt \int _{0}^{1}u^{-HKd}(1-u)^{-HKd} \,du \\ &\quad =\frac{2}{(2\pi )^{d}k^{\frac{d}{2}}}\text{B}(1-HKd,1-HKd) \int_{0} ^{T}t^{(1-2HKd)}\,dt \\ &\quad = \frac{2\varGamma^{2}(1-HKd)}{(2\pi )^{d}k^{\frac{2}{d}}\varGamma (3-HKd)}T ^{2-2HKd}. \end{aligned}$$

This completes the proof. □

### Remark

this proposition implies that the local time of bifractional Brownian motion exists in $L^{2}$ if $HKd<1$. This is consistent with Theorem 2.6 in [3] and Theorem 1 in [12].

## The existence of the renormalized self-intersection local time

In this section, we will prove the existence of the renormalized self-intersection local time of bifractional Brownian motion, which extends the result of Hu and Nualart [7] to bifractional Brownian motion. For more on the existence of the self-intersection local time of bifractional Brownian motion, we can refer to Jiang and Wang [9] and Chen et al. [3].

According to the definition for the self-intersection local time of bifractional Brownian motion and (1.7), we get

3.1 $$\begin{aligned} &L_{\varepsilon }(H,K,T) \\ &\quad = \int^{T}_{0} \int^{t}_{0}p_{\varepsilon } \bigl(B ^{H,K}(t)-B^{H,K}(s) \bigr)\,ds\,dt \\ &\quad =\frac{1}{(2\pi )^{d}} \int^{T}_{0} \int^{t}_{0} \int_{\mathbb{R}^{d}} \exp \biggl\{ i\bigl\langle \xi ,B^{H,K}(t)-B^{H,K}(s) \bigr\rangle - \frac{1}{2}\varepsilon {\vert \xi \vert }^{2} \biggr\} \,d{\xi }\,ds\,dt. \end{aligned}$$

Then, by the independence of $B^{H,K}_{1},\ldots ,B^{H,K}_{d}$, we obtain the mean of the self-intersection local time

3.2 $$\begin{aligned} &\mathbb{E} \bigl[L_{\varepsilon }(H,K,T) \bigr] \\ &\quad =\frac{1}{(2\pi )^{d}} \int^{T}_{0} \int^{t}_{0} \int_{\mathbb{R}^{d}} \mathbb{E}\exp \Biggl\{ i\sum _{m=1}^{d} \bigl(B^{H,K}_{m}(t)-B^{H,K}_{m}(s) \bigr) \xi_{m} \Biggr\} \exp \biggl\{ -\frac{1}{2}\varepsilon { \vert \xi \vert }^{2} \biggr\} d {\xi }\,ds\,dt \\ &\quad =\frac{1}{(2\pi )^{d}} \int^{T}_{0} \int^{t}_{0} \int_{\mathbb{R}^{d}} \prod_{m=1}^{d} \exp \biggl\{ -\frac{1}{2}\operatorname{Var} \bigl( \bigl(B^{H,K}_{m}(t) \\ &\quad \quad {}-B ^{H,K}_{m}(s) \bigr)\xi_{m} \bigr) \biggr\} \exp \biggl\{ -\frac{1}{2}\varepsilon {\vert \xi \vert }^{2} \biggr\} \,d{\xi }\,ds\,dt \\ &\quad =\frac{1}{(2\pi )^{d}} \int^{T}_{0} \int^{t}_{0} \int_{\mathbb{R}^{d}} \prod_{m=1}^{d} \exp \biggl\{ -\frac{1}{2}\vert \xi_{m} \vert ^{2} \bigl(\operatorname{Var} \bigl(B ^{H,K}_{m}(t)-B^{H,K}_{m}(s) \bigr)+\varepsilon \bigr) \biggr\} \,d{\xi }\,ds\,dt \\ &\quad =\frac{1}{(2\pi )^{\frac{d}{2}}} \int^{T}_{0} \int^{t}_{0} \bigl(\operatorname{Var} \bigl(B ^{H,K}_{1}(t)-B^{H,K}_{1}(s) \bigr)+ \varepsilon \bigr)^{-\frac{d}{2}}\,ds\,dt, \end{aligned}$$

and the second moment of the self-intersection local time

$$\begin{aligned} &\mathbb{E} \bigl[L_{\varepsilon }^{2}(H,K,T) \bigr] \\ &\quad =\mathbb{E} \biggl[ \int^{T}_{0} \int^{t}_{0} \int^{T}_{0} \int^{t'}_{0}p _{\varepsilon } \bigl(B^{H,K}(t)-B^{H,K}(s) \bigr) p_{\varepsilon } \bigl(B^{H,K} \bigl(t' \bigr)-B ^{H,K} \bigl(s' \bigr) \bigr)\,ds' \,dt'\,ds\,dt \biggr] \\ &\quad =\frac{1}{(2\pi )^{2d}}\mathbb{E} \biggl[ \int_{\mathcal{T}} \int_{\mathbb{R}^{2d}}\exp \biggl\{ i\bigl\langle \xi ,B^{H,K}(t)-B^{H,K}(s) \bigr\rangle - \frac{1}{2}\varepsilon {\vert \xi \vert }^{2} \biggr\} \\ &\quad \quad {}\times \exp \biggl\{ i\bigl\langle \eta ,B^{H,K} \bigl(t' \bigr)-B^{H,K} \bigl(s' \bigr)\bigr\rangle -\frac{1}{2} \varepsilon { \vert \eta \vert }^{2} \biggr\} \,d{\xi }d{\eta }\,d \tau \biggr] \\ &\quad =\frac{1}{(2\pi )^{2d}} \int_{\mathcal{T}} \int_{\mathbb{R}^{2d}} \exp \biggl\{ -\frac{1}{2}\varepsilon \bigl({ \vert \xi \vert }^{2}+{\vert \eta \vert }^{2} \bigr) \biggr\} \mathbb{E}\exp \Biggl\{ i\sum_{m=1}^{d} \bigl[ \bigl(B^{H,K}_{m}(t)-B ^{H,K}_{m}(s) \bigr)\xi_{m} \\ &\quad \quad {}+ \bigl(B^{H,K}_{m} \bigl(t' \bigr)-B^{H,K}_{m} \bigl(s' \bigr) \bigr) \eta_{m} \bigr] \Biggr\} \,d{\xi }d{\eta }\,d\tau \\ &\quad =\frac{1}{(2\pi )^{2d}} \int_{\mathcal{T}} \int_{\mathbb{R}^{2d}} \exp \biggl\{ -\frac{1}{2}\varepsilon \bigl({ \vert \xi \vert }^{2}+{\vert \eta \vert }^{2} \bigr) \biggr\} \prod_{m=1}^{d}\exp \biggl\{ - \frac{1}{2}\operatorname{Var} \bigl[ \bigl(B^{H,K} _{m}(t)-B^{H,K}_{m}(s) \bigr)\xi_{m} \\ &\quad \quad {}+ \bigl(B^{H,K}_{m} \bigl(t' \bigr)-B^{H,K}_{m} \bigl(s' \bigr) \bigr) \eta_{m} \bigr] \biggr\} \,d{\xi }d{\eta }\,d\tau , \end{aligned}$$

where $\mathcal{T}=\{(s,t,s',t')\mid 0\leq s< t\leq T, 0\leq s'< t'\leq T\}$ and $\tau =(s,t,s',t')$.

Notice that

$$\begin{aligned} &\operatorname{Var} \bigl[ \bigl(B^{H,K}_{m}(t)-B^{H,K}_{m}(s) \bigr)\xi_{m}+ \bigl(B^{H,K}_{m} \bigl(t' \bigr)-B ^{H,K}_{m} \bigl(s' \bigr) \bigr) \eta_{m} \bigr] \\ &\quad =\mathbb{E} \bigl[ \bigl(B^{H,K}_{m}(t)-B^{H,K}_{m}(s) \bigr)\xi_{m}+ \bigl(B^{H,K}_{m} \bigl(t' \bigr)-B ^{H,K}_{m} \bigl(s' \bigr) \bigr) \eta_{m} \bigr]^{2} \\ &\quad =\mathbb{E} \bigl[ \bigl(B^{H,K}_{m}(t)-B^{H,K}_{m}(s) \bigr)^{2}\xi_{m}^{2}+ \bigl(B ^{H,K}_{m} \bigl(t' \bigr)-B^{H,K}_{m} \bigl(s' \bigr) \bigr)^{2}\eta_{m}^{2} \\ & \qquad{}+2\xi_{m}\eta_{m} \bigl(B^{H,K}_{m}(t)-B^{H,K}_{m}(s) \bigr) \bigl(B^{H,K}_{m} \bigl(t' \bigr)-B ^{H,K}_{m} \bigl(s' \bigr) \bigr) \bigr] \\ &\quad =\vert \xi_{m} \vert ^{2}\lambda +\vert \eta_{m} \vert ^{2}\rho +2\xi_{m} \eta_{m}\mu , \end{aligned}$$

where $B^{H,K}_{1}(t)$ denotes a one-dimensional bifractional Brownian motion with Hurst parameters *H* and *K*, *λ* is the variance of $B^{H,K}_{1}(t)-B^{H,K}_{1}(s)$, *ρ* is the variance of $B^{H,K}_{1}(t')-B^{H,K}_{1}(s')$, and *μ* is the covariance of $B^{H,K}_{1}(t)-B^{H,K}_{1}(s)$ and $B^{H,K}_{1}(t')-B^{H,K}_{1}(s')$.

Then the second moment of the random variable $L_{\varepsilon }(H,K,T)$ is

3.3 $$\begin{aligned} &\mathbb{E} \bigl[L_{\varepsilon }^{2}(H,K,T) \bigr] \\ &\quad =\frac{1}{(2\pi )^{2d}} \int_{\mathcal{T}} \int_{\mathbb{R}^{2d}} \prod_{m=1}^{d} \exp \biggl\{ -\frac{1}{2} \bigl[\vert \xi_{m} \vert ^{2}\lambda +\vert \eta_{m} \vert ^{2} \rho +2\xi_{m}\eta_{m}\mu \bigr] \biggr\} \\ &\quad \quad {}\times \exp \biggl\{ - \frac{1}{2}\varepsilon \bigl({\vert \xi \vert }^{2}+{\vert \eta \vert }^{2} \bigr) \biggr\} \,d{\xi }d {\eta }\,d\tau \\ &\quad =\frac{1}{(2\pi )^{2d}} \int_{\mathcal{T}} \int_{\mathbb{R}^{2d}} \exp \biggl\{ -\frac{1}{2} \bigl(\vert \xi \vert ^{2}\lambda +\vert \eta \vert ^{2}\rho +2\langle \xi ,\eta \rangle \mu \bigr) \biggr\} \\ &\quad \quad {}\times \exp \biggl\{ -\frac{1}{2}\varepsilon \bigl({ \vert \xi \vert }^{2}+ {\vert \eta \vert }^{2} \bigr) \biggr\} \,d{\xi }d{\eta }\,d\tau \\ &\quad =\frac{1}{(2\pi )^{d}} \int_{\mathcal{T}} \bigl[(\lambda +\varepsilon ) ( \rho +\varepsilon )- \mu^{2} \bigr]^{-\frac{d}{2}}\,d\tau . \end{aligned}$$

Based on $\mathbb{E}[L_{\varepsilon }(H,K,T)]$ and $\mathbb{E}[L_{ \varepsilon }^{2}(H,K,T)]$, it follows that

3.4 $$\begin{aligned} \mathbb{E} & \bigl(L_{\varepsilon_{1}}(H,K,T)-\mathbb{E} \bigl[L_{\varepsilon _{1}}(H,K,T) \bigr] \bigr) \bigl(L_{\varepsilon_{2}}(H,K,T)- \mathbb{E} \bigl[L_{ \varepsilon_{2}}(H,K,T) \bigr] \bigr) \\ &=\mathbb{E} \bigl(L_{\varepsilon_{1}}(H,K,T)L_{\varepsilon_{2}}(H,K,T) \bigr)- \bigl( \mathbb{E} \bigl[L_{\varepsilon_{1}}(H,K,T) \bigr]\mathbb{E} \bigl[L_{ \varepsilon_{2}}(H,K,T) \bigr] \bigr) \\ &=\frac{1}{(2\pi )^{d}} \int_{\mathcal{T}} \bigl[ \bigl[(\lambda +\varepsilon _{1}) ( \rho +\varepsilon_{2})-\mu^{2} \bigr]^{-\frac{d}{2}} -({ \lambda }+ \varepsilon_{1})^{-\frac{d}{2}}({\rho }+ \varepsilon_{2})^{- \frac{d}{2}} \bigr]\,d\tau . \end{aligned}$$

By equality (3.4), we have

$$\begin{aligned} &\lim_{\varepsilon_{1},\varepsilon_{2}\rightarrow 0} \mathbb{E} \bigl(L _{\varepsilon_{1}}(H,K,T)- \mathbb{E} \bigl[L_{\varepsilon_{1}}(H,K,T) \bigr] \bigr) \bigl(L_{\varepsilon_{2}}(H,K,T)- \mathbb{E} \bigl[L_{\varepsilon_{2}}(H,K,T) \bigr] \bigr) \\ &\quad =\lim_{\varepsilon_{1},\varepsilon_{2}\rightarrow 0}\frac{1}{(2 \pi )^{d}} \int_{\mathcal{T}} \bigl[ \bigl[(\lambda +\varepsilon_{1}) ( \rho + \varepsilon_{2})-\mu^{2} \bigr]^{-\frac{d}{2}} -({ \lambda }+\varepsilon_{1})^{- \frac{d}{2}}({\rho }+\varepsilon_{2})^{-\frac{d}{2}} \bigr]\,d\tau \\ &\quad =\frac{1}{(2\pi )^{d}} \int_{\mathcal{T}} \bigl[ \bigl(\lambda \rho -\mu^{2} \bigr)^{- \frac{d}{2}}-(\lambda \rho )^{-\frac{d}{2}} \bigr]\,d\tau . \end{aligned}$$

Therefore, the limit of

3.5 $$\begin{aligned} \mathbb{E} & \bigl(L_{\varepsilon_{1}}(H,K,T)-\mathbb{E} \bigl[L_{\varepsilon _{1}}(H,K,T) \bigr] \bigr) \bigl(L_{\varepsilon_{2}}(H,K,T)- \mathbb{E} \bigl[L_{ \varepsilon_{2}}(H,K,T) \bigr] \bigr), \end{aligned}$$

as $\varepsilon_{1}$, $\varepsilon_{2}$ tend to 0, exists if and only if $\int_{\mathcal{T}}[(\lambda \rho -\mu^{2})^{-\frac{d}{2}}-(\lambda \rho )^{-\frac{d}{2}}]\,d\tau $.

By Loève’s criterion of mean-square convergence, we know that a necessary and sufficient condition for the convergence of $L_{\varepsilon }(H,K,T)-\mathbb{E}[L_{\varepsilon }(H,K,T)] $ in $L^{2}$ is the existence of finite limit of (3.5) as $\varepsilon_{1}$, $\varepsilon_{2}$ tend to 0. Consequently, a necessary and sufficient condition for the convergence of $L_{\varepsilon }(H,K,T)-\mathbb{E}[L _{\varepsilon }(H,K,T)]$ in $L^{2}$ is that

3.6 $$\begin{aligned} \varXi_{T}:= \int_{\mathcal{T}} \bigl[ \bigl(\lambda \rho -\mu^{2} \bigr)^{-\frac{d}{2}}-( \lambda \rho )^{-\frac{d}{2}} \bigr]\,d\tau < +\infty . \end{aligned}$$

For notation and simplicity along the paper, we will denote $\delta =\lambda \rho -\mu^{2}$ and $\varTheta =\delta^{-\frac{d}{2}}-( \lambda \rho )^{-\frac{d}{2}}$, then the last inequality is rewritten as

3.7 $$\begin{aligned} \varXi_{T}= \int_{\mathcal{T}}\varTheta \,d\tau < +\infty . \end{aligned}$$

For simplicity, we give some symbols. The region $\mathcal{T}=\{(s,t,s',t')\mid 0\leq s< t\leq T,0\leq s'< t'\leq T\}$ is decomposed as follows:

$$ \mathcal{T} \cap \bigl\{ s< s' \bigr\} =\mathcal{T}_{1} \cup \mathcal{T}_{2}\cup\mathcal{T}_{3}, $$

where

$$\begin{aligned}& \mathcal{T}_{1}= \bigl\{ \bigl(s,t,s',t' \bigr)\mid 0\leq s< s'< t< t'\leq T \bigr\} , \\& \mathcal{T}_{2}= \bigl\{ \bigl(s,t,s',t' \bigr)\mid 0\leq s< s'< t'< t\leq T \bigr\} , \\& \mathcal{T}_{3}= \bigl\{ \bigl(s,t,s',t' \bigr)\mid 0\leq s< t< s'< t'\leq T \bigr\} . \end{aligned}$$

Then, for $(s,t,s',t')\in \mathcal{T}$, we can consider the following three cases:

(i) If $(s,t,s',t')\in \mathcal{T}_{1}$, denoting $a=s'-s$, $b=t-s'$, $c=t'-t$, and $e=s$, we have
  $$\begin{aligned}& \lambda =\lambda_{1} :=(a+b)^{2HK}, \qquad \rho = \rho_{1}:=(b+c)^{2HK}, \\& \mu =\mu_{1} :=\frac{1}{2^{K}} \bigl[ \bigl[(e+a+b)^{2H}+(e+a+b+c)^{2H} \bigr]^{K}- \bigl[(e+a+b)^{2H}+(e+a)^{2H} \bigr]^{K} \\& \hphantom{\mu =\mu_{1} :=} - \bigl[e^{2H}+(e+a+b+c)^{2H} \bigr]^{K}+ \bigl[e^{2H}+(e+a)^{2H} \bigr]^{K} \bigr] \\& \hphantom{\mu =\mu_{1} :=}+\frac{1}{2^{K}} \bigl[b^{2HK}-c^{2HK}-a^{2HK}+(a+b+c)^{2HK} \bigr]. \end{aligned}$$
(ii) If $(s,t,s',t')\in \mathcal{T}_{2}$, denoting $a=s'-s$, $b=t'-s'$, $c=t-t'$, and $e=s$, we have
  $$\begin{aligned}& \lambda =\lambda_{2} :=(a+b+c)^{2HK}, \qquad \rho = \rho_{2}:=b^{2HK}, \\& \mu =\mu_{2} :=\frac{1}{2^{K}} \bigl[ \bigl[(e+a+b+c)^{2H}+(e+a+b)^{2H} \bigr]^{K} \\& \hphantom{\mu =\mu_{1} :=}- \bigl[(e+a+b+c)^{2H}+(e+a)^{2H} \bigr]^{K} \\& \hphantom{\mu =\mu_{1} :=}- \bigl[e^{2H}+(e+a+b)^{2H} \bigr]^{K}+ \bigl[e^{2H}+(e+a)^{2H} \bigr]^{K} \bigr] \\& \hphantom{\mu =\mu_{1} :=}+\frac{1}{2^{K}} \bigl[(b+c)^{2HK}-c^{2HK}-a^{2HK}+(a+b)^{2HK} \bigr]. \end{aligned}$$
(iii) If $(s,t,s',t')\in \mathcal{T}_{3}$, denoting $a=t-s$, $b=s'-t$, $c=t'-s'$, and $e=s$, we have
  $$\begin{aligned}& \lambda =\lambda_{3} :=a^{2HK}, \qquad \rho = \rho_{3}:=c^{2HK}, \\& \mu =\mu_{3} :=\frac{1}{2^{K}} \bigl[ \bigl[(e+a)^{2H}+(e+a+b+c)^{2H} \bigr]^{K}- \bigl[(e+a)^{2H}+(e+a+b)^{2H} \bigr]^{K} \\& \hphantom{\mu =\mu_{1} :=}- \bigl[e^{2H}+(e+a+b+c)^{2H} \bigr]^{K}+ \bigl[e^{2H}+(e+a+b)^{2H} \bigr]^{K} \bigr] \\& \hphantom{\mu =\mu_{1} :=} +\frac{1}{2^{K}} \bigl[\vert a+b+c \vert ^{2HK}- \vert a+b \vert ^{2HK}-\vert b+c \vert ^{2HK}+b^{2HK} \bigr]. \end{aligned}$$

Meanwhile, we set $\delta_{i}=\lambda_{i}\rho_{i}-\mu^{2}_{i}$ and $\varTheta_{i}=\delta_{i}^{-\frac{d}{2}}-(\lambda_{i}\rho_{i})^{- \frac{d}{2}}$ for $i=1,2,3$.

For $\mu_{3}$, we can get the following upper bounded by the basic inequalities.

### Lemma 3.1

*For* $\mu_{3}$ *defined in case* (iii), *there exists a constant* *k* *such that*

3.8 $$\begin{aligned} \mu_{3}\leq kb^{2HK-2}ac. \end{aligned}$$

### Proof

For the convenience of calculation, we set $\mu_{3}:= \Delta_{3,1}+\Delta_{3,2}$, where

$$\begin{aligned} \Delta_{3,1}:=\frac{1}{2^{K}} \bigl[\vert a+b+c \vert ^{2HK}-\vert a+b \vert ^{2HK}-\vert b+c \vert ^{2HK}+\vert b \vert ^{2HK} \bigr] \end{aligned}$$

and

$$\begin{aligned} \Delta_{3,2} &:=\frac{1}{2^{K}} \bigl[ \bigl[(e+a)^{2H}+(e+a+b+c)^{2H} \bigr]^{K}- \bigl[(e+a)^{2H}+(e+a+b)^{2H} \bigr]^{K} \\ & \quad {}- \bigl[e^{2H}+(e+a+b+c)^{2H} \bigr]^{K}+ \bigl[e^{2H}+(e+a+b)^{2H} \bigr]^{K} \bigr]. \end{aligned}$$

For the term $\Delta_{3,1}$, we easily get $\Delta_{3,1} \leq kacb ^{2HK-2}$.

Now, we consider the term $\Delta_{3,2}$. Let $f(t)=[t^{2H}+(e+a+b+c)^{2H}]^{K}-[t ^{2H}+(e+a+b)^{2H}]^{K}$. Then we have

$$\begin{aligned} \frac{df(t)}{\,dt}=2HKt^{2H-1} \bigl\{ \bigl[t^{2H}+(e+a+b+c)^{2H} \bigr]^{K-1}- \bigl[t^{2H}+(e+a+b)^{2H} \bigr]^{K-1} \bigr\} . \end{aligned}$$

So, by the mean theorem, there exist $\xi \in (e, e+a)$ and $\eta \in (e+a+b, e+a+b+c)$ such that

3.9 $$\begin{aligned} \Delta_{3,2} &=\frac{1}{2^{K}} \int_{e}^{e+a}2HKt^{2H-1} \bigl\{ \bigl[t^{2H}+(e+a+b+c)^{2H} \bigr]^{K-1}- \bigl[t ^{2H}+(e+a+b)^{2H} \bigr]^{K-1} \bigr\} \,dt \\ &\leq \frac{1}{2^{K}} \bigl\vert 2HKa\xi^{2H-1} \bigl\{ \bigl[ \xi^{2H}+(e+a+b+c)^{2H} \bigr]^{K-1}- \bigl[ \xi^{2H}+(e+a+b)^{2H} \bigr]^{K-1} \bigr\} \bigr\vert \\ &=\frac{1}{2^{K}} \bigl\vert 4H^{2}K(K-1) \bigr\vert \xi^{2H-1}\eta^{2H-1} \bigl[\xi^{2H}+\eta ^{2H} \bigr]^{K-2}ac \\ &=kac\eta^{2HK-2}\leq kac(e+a+b)^{2HK-2} \\ &\leq kacb^{2HK-2}, \end{aligned}$$

where we used the fact that $\xi^{2H}+\eta^{2H}\geq \xi^{2H\alpha } \eta^{2H\eta }$, $\alpha =\frac{1-2H}{2H(K-2)}, \beta ={1- \frac{1-2H}{2H(K-2)}}$. Further,

$$\begin{aligned} \xi^{2H-1}\eta^{2H-1} \bigl[\xi^{2H}+\eta^{2H} \bigr]^{K-2}\leq \xi^{2H-1}\eta ^{2H-1} \bigl[ \xi^{2H\alpha }\eta^{2H\eta } \bigr]^{K-2}\leq \eta^{2HK-2}. \end{aligned}$$

Combining the upper bounds of $\Delta_{3,1}$ and $\Delta_{3,2}$, we get that $\mu_{3} \leq kb^{2HK-2}ac$. □

The following lemma provides some useful inequalities for the proof of Theorem 1.2.

### Lemma 3.2

*Following the decomposition of the region* $\mathcal{T}$, *there exists a constant* *k* *such that*

(i) 3.10 $$\begin{aligned} \delta_{1}\geq k \bigl[(a+b)^{2HK}c^{2HK}+(b+c)^{2HK} a^{2HK} \bigr], \end{aligned}$$
(ii) *for* $i=2,3$,
  3.11 $$\begin{aligned} \delta_{i}\geq k\lambda_{i} \rho_{i}. \end{aligned}$$

### Proof

For case (i), we denote $\bar{a}=\operatorname{Var}(B^{H,K}_{1}(s')-B ^{H,K}_{1}(s))$, $\bar{b}=\operatorname{Var}(B^{H,K}_{1}(t)-B^{H,K}_{1}(s'))$, $\bar{c}=\operatorname{Var}(B^{H,K}_{1}(t')-B^{H,K}_{1}(t))$. On the one hand, by the local nondeterminism (2.2), we have, for all *u* and *v*,

3.12 $$\begin{aligned} &\operatorname{Var} \bigl(u \bigl(B^{H,K}_{1}(t)-B^{H,K}_{1}(s) \bigr)+v \bigl(B^{H,K}_{1} \bigl(t' \bigr)-B^{H,K} _{1} \bigl(s' \bigr) \bigr) \bigr) \\ &\quad =\operatorname{Var} \bigl(u \bigl(B^{H,K}_{1} \bigl(s' \bigr)-B^{H,K}_{1}(s) \bigr)+(u+v) \bigl(B^{H,K}_{1}(t)-B ^{H,K}_{1} \bigl(s' \bigr) \bigr) \\ & \qquad {}+v \bigl(B^{H,K}_{1} \bigl(t' \bigr)-B^{H,K}_{1}(t) \bigr) \bigr) \\ &\quad \geq k \bigl[\bar{a}u^{2}+\bar{b}(u+v)^{2}+ \bar{c}v^{2} \bigr]. \end{aligned}$$

On the other hand, by using inequality (1.2), we get

3.13 $$\begin{aligned} &\operatorname{Var} \bigl(u \bigl(B^{H,K}_{1}(t)-B^{H,K}_{1}(s) \bigr)+v \bigl(B^{H,K}_{1} \bigl(t' \bigr)-B^{H,K} _{1} \bigl(s' \bigr) \bigr) \bigr) \\ &\quad =\mathbb{E} \bigl[u \bigl(B^{H,K}_{1}(t)-B^{H,K}_{1}(s) \bigr)+v \bigl(B^{H,K}_{1} \bigl(t' \bigr)-B ^{H,K}_{1} \bigl(s' \bigr) \bigr) \bigr]^{2} \\ &\quad =u^{2}\mathbb{E} \bigl(B^{H,K}_{1}(t)-B^{H,K}_{1}(s) \bigr)^{2}+v^{2}\mathbb{E} \bigl(B ^{H,K}_{1} \bigl(t' \bigr)-B^{H,K}_{1} \bigl(s' \bigr) \bigr)^{2} \\ & \qquad {}+2uv \mathbb{E} \bigl[ \bigl(B^{H,K}_{1}(t)-B^{H,K}_{1}(s) \bigr) \bigl(B^{H,K}_{1} \bigl(t' \bigr)-B^{H,K} _{1} \bigl(s' \bigr) \bigr) \bigr] \\ &\quad =\lambda_{1} u^{2}+\rho_{1} v^{2}+2 \mu_{1}uv. \end{aligned}$$

Thus, through combining (3.12) with (3.13), it is easy to get

$$\begin{aligned} \lambda_{1} u^{2}+\rho_{1} v^{2}+2 \mu_{1}uv \geq k \bigl[\bar{a}u^{2}+ \bar{b}(u+v)^{2}+ \bar{c}v^{2} \bigr]. \end{aligned}$$

This implies that

$$\begin{aligned} (\lambda_{1}-k\bar{a}-k\bar{b})u^{2}+2(\mu_{1}-k \bar{b})uv+(\rho_{1}-k \bar{b}-k\bar{c})v^{2}\geq 0. \end{aligned}$$

Therefore, $(\lambda_{1}-k\bar{a}-k\bar{b})(\rho_{1}-k\bar{b}-k \bar{c})-(\mu_{1}-k\bar{b})^{2}\geq 0$.

By calculating, we have

$$\begin{aligned} \delta_{1} &=\lambda_{1}\rho_{1}- \mu_{1}^{2} \\ &\geq \lambda_{1} k(\bar{b}+\bar{c})+k\rho_{1}(\bar{a}+ \bar{b})-2k\mu _{1} \bar{b}-k^{2}(\bar{a}\bar{b}+\bar{b} \bar{c}+\bar{a}\bar{c}). \end{aligned}$$

According to the inequality $\mu_{1}^{2}\leq \lambda_{1}\rho_{1}$, it is easy to get $\mu_{1}\leq \sqrt{\lambda_{1}\rho_{1}}\leq \frac{1}{2}(\lambda_{1}+\rho_{1})$. Furthermore, we get that $\delta_{1}$ has the lower bound as follows:

$$\begin{aligned} \delta_{1}\geq k(\lambda_{1} \bar{c}+\rho_{1} \bar{a})-k^{2}(\bar{a} \bar{b}+\bar{b}\bar{c}+\bar{a}\bar{c}). \end{aligned}$$

Meanwhile, based on $C_{r}$-inequality and the denotes *ā*, *b̄*, *c̄*, we know that $\lambda_{1}\leq 2(\bar{a}+\bar{b})= 2( {a}^{2HK}+{b}^{2HK})$, $\rho_{1}\leq 2(\bar{b}+\bar{c})= 2({b}^{2HK}+ {c}^{2HK})$, then

$$\begin{aligned} \lambda_{1} \bar{c}+\rho_{1} \bar{a}\leq 2(\bar{a}+ \bar{b})\bar{c}+2( \bar{b}+\bar{c})\bar{a} \leq 4(\bar{a}\bar{b}+\bar{b}\bar{c}+ \bar{a} \bar{c}). \end{aligned}$$

Furthermore, we can choose $k\in (0,4)$ satisfying inequality (2.2) such that the following inequality holds:

$$\begin{aligned} \delta_{1} &\geq \biggl(k-\frac{k^{2}}{4} \biggr) ( \lambda_{1} \bar{c}+\rho_{1} \bar{a}) \\ &=\frac{(4-k)k}{4}(\lambda_{1} \bar{c}+\rho_{1} \bar{a}) \\ &\geq k \bigl[(\bar{a}+\bar{b})\bar{c}+(\bar{b}+\bar{c})\bar{a} \bigr] \\ &\geq k \bigl[ \bigl({a}^{2HK}+{b}^{2HK} \bigr){c}^{2HK}+ \bigl({b}^{2HK}+{c}^{2HK} \bigr){a}^{2HK} \bigr] \\ &\geq k \bigl[(a+b)^{2HK}{c}^{2HK}+(b+c)^{2HK}{a}^{2HK} \bigr], \end{aligned}$$

where we used the fact $(a+b)^{2HK}\leq k({a}^{2HK}+{b}^{2HK})$.

Next, we prove case (ii). For $i=2$, denote $\bar{a}=\operatorname{Var}(B^{H,K} _{1}(s')-B^{H,K}_{1}(s))$, $\bar{b}=\operatorname{Var}(B^{H,K}_{1}(t')-B^{H,K} _{1}(s'))$, $\bar{c}=\operatorname{Var}(B^{H,K}_{1}(t)-B^{H,K}_{1}(t'))$. By (2.2), we get that $\lambda_{2}\geq k(\bar{a}+\bar{b}+ \bar{c})$, $\rho_{2}\geq k\bar{b}$, and for all *u* and *v*, we have

3.14 $$\begin{aligned} &\operatorname{Var} \bigl(u \bigl(B^{H,K}_{1}(t)-B^{H,K}_{1}(s) \bigr)+v \bigl(B^{H,K}_{1} \bigl(t' \bigr)-B^{H,K} _{1} \bigl(s' \bigr) \bigr) \bigr) \\ &\quad =\mathbb{E} \bigl[u \bigl(B^{H,K}_{1}(t)-B^{H,K}_{1}(s) \bigr)+v \bigl(B^{H,K}_{1} \bigl(t' \bigr)-B ^{H,K}_{1} \bigl(s' \bigr) \bigr) \bigr]^{2} \\ &\quad \geq k \bigl(\bar{b}v^{2}+(\bar{a}+\bar{b}+ \bar{c})u^{2} \bigr). \end{aligned}$$

On the other hand,

3.15 $$\begin{aligned} &\operatorname{Var} \bigl(u \bigl(B^{H,K}_{1}(t)-B^{H,K}_{1}(s) \bigr)+v \bigl(B^{H,K}_{1} \bigl(t' \bigr)-B^{H,K} _{1} \bigl(s' \bigr) \bigr) \bigr) \\ &\quad =\mathbb{E} \bigl[u \bigl(B^{H,K}_{1}(t)-B^{H,K}_{1}(s) \bigr)+v \bigl(B^{H,K}_{1} \bigl(t' \bigr)-B ^{H,K}_{1} \bigl(s' \bigr) \bigr) \bigr]^{2} \\ &\quad =u^{2}\mathbb{E} \bigl(B^{H,K}_{1}(t)-B^{H,K}_{1}(s) \bigr)^{2}+v^{2}\mathbb{E} \bigl(B ^{H,K}_{1} \bigl(t' \bigr)-B^{H,K}_{1} \bigl(s' \bigr) \bigr)^{2} \\ & \quad \quad {}+2uv \mathbb{E} \bigl[ \bigl(B^{H,K}_{1}(t)-B^{H,K}_{1}(s) \bigr) \bigl(B^{H,K}_{1} \bigl(t' \bigr)-B^{H,K} _{1} \bigl(s' \bigr) \bigr) \bigr] \\ &\quad =\lambda_{2} u^{2}+\rho_{2} v^{2}+2 \mu_{2}uv. \end{aligned}$$

Then, we have the following inequality by combining (3.14) with (3.15):

$$\begin{aligned} \lambda_{2} u^{2}+2\mu_{2} uv+\rho_{2} v^{2}\geq k \bigl(\bar{b}v^{2}+( \bar{a}+\bar{b}+ \bar{c})u^{2} \bigr). \end{aligned}$$

Thus, it is easy to see

$$\begin{aligned} \bigl[\lambda_{2}-k(\bar{a}+\bar{b}+\bar{c}) \bigr]( \rho_{2}-k\bar{b})-\mu_{2} ^{2}\geq 0. \end{aligned}$$

Furthermore, according to the definition of *δ*, we can get

$$\begin{aligned} \delta_{2} &=\lambda_{2}\rho_{2}- \mu_{2}^{2} \\ &\geq k\lambda_{2}\bar{b}+k\rho_{2}(\bar{a}+\bar{b}+ \bar{c})-k^{2}( \bar{a}+\bar{b}+\bar{c})\bar{b} \\ &\geq k\lambda_{2}\rho_{2}. \end{aligned}$$

For $i=3$, denote $\bar{a}=\operatorname{Var}(B^{H,K}_{1}(t)-B^{H,K}_{1}(s))$, $\bar{b}=\operatorname{Var}(B^{H,K}_{1}(s')-B^{H,K}_{1}(t))$, $\bar{c}=\operatorname{Var}(B ^{H,K}_{1}(t')-B^{H,K}_{1}(s'))$. It is easy to get $\lambda_{3}= \bar{a}$ and $\rho_{3}=\bar{c}$. Meanwhile, by the local nondeterminism (2.2), for all *u* and *v*,

3.16 $$\begin{aligned} \operatorname{Var} \bigl(u \bigl(B^{H,K}_{1}(t)-B^{H,K}_{1}(s) \bigr)+v \bigl(B^{H,K}_{1} \bigl(t' \bigr)-B^{H,K} _{1} \bigl(s' \bigr) \bigr) \bigr) \geq k \bigl(\bar{a}u^{2}+\bar{c}v^{2} \bigr) \end{aligned}$$

and

3.17 $$\begin{aligned} \operatorname{Var} \bigl(u \bigl(B^{H,K}_{1}(t)-B^{H,K}_{1}(s) \bigr)+v \bigl(B^{H,K}_{1} \bigl(t' \bigr)-B^{H,K} _{1} \bigl(s' \bigr) \bigr) \bigr)= \lambda_{3} u^{2}+\rho_{3} v^{2}+2 \mu_{3}uv. \end{aligned}$$

Then, it follows from combining (3.16) with (3.17) that

$$\begin{aligned} \lambda_{3} u^{2}+2\mu_{3} uv+ \rho_{3} v^{2}\geq k \bigl(\bar{a}u^{2}+ \bar{c}v^{2} \bigr). \end{aligned}$$

Thus,

$$\begin{aligned} (\lambda_{3}-k\bar{a}) (\rho_{3}-k\bar{c})- \mu_{3}^{2}\geq 0. \end{aligned}$$

Therefore, we get

$$\begin{aligned} \delta_{3}=\lambda_{3}\rho_{3}- \mu_{3}^{2}\geq k\lambda_{3} \bar{c}+k \rho_{3} \bar{a}-k^{2} \bar{a}\bar{c} \geq k \lambda_{3}\rho_{3}. \end{aligned}$$

Thus, the proof of Lemma 3.2 is completed. □

By Lemma 3.2 and $\delta_{i}$, $\varTheta_{i}$, $i=1,2,3$, defined above, we can get the following result.

### Lemma 3.3

*For* $i=2,3$, *there exists a constant* *k* *such that*

3.18 $$\begin{aligned} \varTheta_{i}\leq k\mu^{2}_{i}( \lambda_{i}\rho_{i})^{-\frac{d}{2}-1} \end{aligned}$$

*and*

3.19 $$\begin{aligned} \varTheta_{i}\leq k(\lambda_{i} \rho_{i})^{-\frac{d}{2}}. \end{aligned}$$

The proof of this lemma can be found in Hu and Nualart [7].

For proving Theorem 1.2, we will make use of the following elementary lemma.

### Lemma 3.4

*Let* $\varXi_{T}$ *be defined by* (3.7). *Then* $\varXi_{T}<+\infty $ *if and only if* $HKd<\frac{3}{2}$.

### Proof

The proof will be done in two steps.

*Step* 1. We give the proof of the sufficient condition, that is, if $HKd<\frac{3}{2}$, we claim that

3.20 $$\begin{aligned} \int_{[0,T]^{3}}\varTheta_{i}\,da\,db\,dc< +\infty , \quad i=1,2,3. \end{aligned}$$

We split the proof into three cases for the value of *i*.

For $i=1$, it is easy to get from (3.10)

3.21 $$\begin{aligned} \delta_{1} &\geq k \bigl[(a+b)^{2HK}c^{2HK}+(b +c)^{2HK} a^{2HK} \bigr] \\ &\geq k(a+b)^{HK}(b+c)^{HK}a^{HK}c^{HK} \\ &\geq k(abc)^{\frac{4}{3}HK} \end{aligned}$$

and

3.22 $$\begin{aligned} \lambda_{1}\rho_{1}\geq k(a+b)^{2HK}(b+c)^{2HK}\geq (ab c)^{ \frac{4}{3}HK}. \end{aligned}$$

Thus, the result $\int_{[0,T]^{3}}\varTheta_{1}\,da\,db\,dc<+\infty $ can be obtained by combining the last two inequalities (3.21) and (3.22) for $i=1$.

For $i=2$, we decompose the region $\mathcal{T}_{2}$ as $\mathcal{T}_{2}=\{b\geq \eta_{1} a\}\cup \{b\geq \eta_{2} c\}\cup \{b< \eta_{1} a, b<\eta_{2} c\}$ for some fixed but arbitrary $\eta_{1}>0$ and $\eta_{2}>0$.

When $b<\eta_{1} a$ and $b<\eta_{2} c$, following the definition of $\mu_{2}$, we set

$$\begin{aligned} \mu_{2}:=\Delta_{2,1}+\Delta_{2,2}, \end{aligned}$$

where

$$\begin{aligned} \Delta_{2,1} &:=\frac{1}{2^{K}} \bigl[ \bigl[(e+a+b+c)^{2H}+(e+a+b)^{2H} \bigr]^{K}- \bigl[(e+a+b+c)^{2H}+(e+a)^{2H} \bigr]^{K} \\ & \quad{} - \bigl[e^{2H}+(e+a+b)^{2H} \bigr]^{K}+ \bigl[e^{2H}+(e+a)^{2H} \bigr]^{K} \bigr] \end{aligned}$$

and

$$ \Delta_{2,2}:=\frac{1}{2^{K}} \bigl[(b+c)^{2HK}-c^{2HK}-a^{2HK}+(a+b)^{2HK} \bigr]. $$

It is easy to get

$$\begin{aligned} \Delta_{2,2} &=\frac{1}{2^{K}} \bigl[\vert a+b \vert ^{2HK}-\vert a \vert ^{2HK}+\vert b+c \vert ^{2HK}-c^{2HK} \bigr] \\ &=\frac{1}{2^{K}}2HK \int_{0}^{b}(a+x)^{2HK-1}+(c+x)^{2HK-1} \,dx \\ &\leq k \bigl(a^{2HK-1}+c^{2HK-1} \bigr)b. \end{aligned}$$

Next, we consider $\Delta_{2,1}$. We know that $\Delta_{2,1}$ can be rewritten as follows:

$$ \Delta_{2,1}=\frac{1}{2^{K}} \bigl[ \bigl(t^{2H}+t^{\prime{2H}} \bigr)^{K}- \bigl(t^{2H}+s^{\prime 2H} \bigr)^{K}- \bigl(s ^{2H}+t^{\prime 2H} \bigr)^{K}+ \bigl(s^{2H}+s^{\prime 2H} \bigr)^{K} \bigr]. $$

Let $f(t,x)=(t^{2H}+x^{2H})^{K}$, by differential we get

$$\begin{aligned} \frac{d}{\,dx}f(t,x) =K \bigl(t^{2H}+x^{2H} \bigr)^{K-1}2Hx^{2H-1}\leq Kx^{2HK-2H}x^{2H-1}=Kx^{2HK-1}. \end{aligned}$$

By the mean theorem, there exists $\xi \in (s',t')$ such that

3.23 $$\begin{aligned} \bigl(t^{2H}+t^{\prime 2H} \bigr)^{K}- \bigl(t^{2H}+s^{\prime 2H} \bigr)^{K} &\leq K \bigl(t^{2H}+\xi^{2H} \bigr)^{K-1}2H \xi^{2H-1} \bigl(t'-s' \bigr) \\ &\leq K\xi^{2HK-1} \bigl(t'-s' \bigr). \end{aligned}$$

If $2HK-1\leq 0$, by $a\leq e+a= s'\leq \xi \leq t'= (e+a+b)$, the last inequality of (3.23) is bounded by

$$\begin{aligned} K(e+a)^{2HK-1}b\leq Ka^{2HK-1}b\leq K \bigl(a^{2HK-1}+c^{2HK-1} \bigr)b. \end{aligned}$$

Then there exists a constant *k* such that

$$\begin{aligned} \Delta_{2,1} &=\frac{1}{2^{K}} \bigl[ \bigl(t^{2H}+t^{\prime 2H} \bigr)^{K}- \bigl(t^{2H}+s^{\prime 2H} \bigr)^{K}- \bigl(s ^{2H}+t^{\prime 2H} \bigr)^{K}+ \bigl(s^{2H}+s^{\prime 2H} \bigr)^{K} \bigr] \\ &\leq k \bigl[ \bigl(t^{2H}+t^{\prime 2H} \bigr)^{K}- \bigl(t^{2H}+s^{\prime 2H} \bigr)^{K} \bigr] \\ &\leq k \bigl(a^{2HK-1}+c^{2HK-1} \bigr)b. \end{aligned}$$

By combining $\Delta_{2,1}$ with $\Delta_{2,2}$, we have $\mu_{2} \leq k(a^{2HK-1}+c^{2HK-1})b$.

If $2HK-1>0$, we have

3.24 $$\begin{aligned} \Delta_{2,1} &=\frac{1}{2^{K}} \bigl[ \bigl(t^{2H}+t^{\prime 2H} \bigr)^{K}- \bigl(t^{2H}+s^{\prime 2H} \bigr)^{K}- \bigl(s ^{2H}+t^{\prime 2H} \bigr)^{K}+ \bigl(s^{2H}+s^{\prime 2H} \bigr)^{K} \bigr] \\ &\leq \frac{1}{2^{K}}2HK \int_{s'}^{t'}x^{2H-1} \bigl[ \bigl(t^{2H}+x^{2H} \bigr)^{K-1}- \bigl(s ^{2H}+x^{2H} \bigr)^{K-1} \bigr]\,dx \\ &\leq \frac{1}{2^{K}}2HK \int_{0}^{T}x^{2H-1} \bigl[ \bigl(t^{2H}+x^{2H} \bigr)^{K-1}- \bigl(s ^{2H}+x^{2H} \bigr)^{K-1} \bigr]\,dx \\ &< 0. \end{aligned}$$

It follows that $\mu_{2}\leq k(a^{2HK-1}+c^{2HK-1})b$.

Using (3.18) of Lemma 3.3, we have

$$\begin{aligned} \varXi_{T} &= \int_{{b< \eta_{1} a},{b< \eta_{2} c}}\varTheta_{2}\,da\,db\,dc \\ &\leq \int_{{b< \eta_{1} a},{b< \eta_{2} c}}k\mu_{2}^{2}( \lambda_{2} \rho_{2})^{-\frac{d}{2}-1}\,da\,db\,dc \\ &\leq \int_{{b< \eta_{1} a},{b< \eta_{2} c}}k \bigl(a^{2HK-1}+c^{2HK-1} \bigr)^{2}b ^{2} \bigl((a+b+c)^{2HK}b^{2HK} \bigr)^{-\frac{d}{2}-1}\,da\,db\,dc \\ &\leq k \int_{{b< \eta_{1} a},{b< \eta_{2} c}} \bigl(a^{4HK-2}+c^{4HK-2} \bigr) (a+b+c)^{-HKd-2HK}b ^{2-HKd-2HK}\,da\,db\,dc \\ &\leq k \int_{{b< \eta_{1} a},{b< \eta_{2} c}} \bigl(a^{(2-\frac{d}{3})HK}b ^{\frac{dHK}{3}}+c^{(2-\frac{d}{3})HK}b^{\frac{dHK}{3}} \bigr) (a+b+c)^{-HKd-2HK}b ^{-HKd}\,da\,db\,dc \\ &\leq k \int_{[0,T]^{3}}a^{(2-\frac{d}{3})HK}b^{\frac{dHK}{3}}(a+b+c)^{-HKd-2HK}b ^{-HKd}\,da\,db\,dc \\ &\leq k \int_{[0,T]^{3}}a^{-\frac{2dHK}{3}}b^{-\frac{2dHK}{3}}c^{- \frac{2dHK}{3}}\,da \,db\,dc< + \infty . \end{aligned}$$

For the case $b\geq \eta_{2} a$, using inequality (3.19) of Lemma 3.3, it is easy to get

3.25 $$\begin{aligned} \varXi_{T} &= \int_{b\geq \eta_{2} a}\varTheta_{2}\,da\,db\,dc \\ &\leq k \int_{b\geq \eta_{2} a}(\lambda_{2}\rho_{2})^{-\frac{d}{2}} \,da\,db\,dc \\ &=k \int_{b\geq \eta_{2} a} \bigl[b^{2HK}(a+b+c)^{2HK} \bigr]^{-\frac{d}{2}}\,da\,db\,dc \\ &=k \int_{b\geq \eta_{2} a}\frac{1}{b^{HKd}(a+b+c)^{HKd}}\,da\,db\,dc. \end{aligned}$$

If $HKd<1$, the last integral of (3.25) is finite.

If $HK\,d\geq 1$, the last integral of (3.25) is written by

$$\begin{aligned} \varXi_{T} &\leq k \int_{[0,T]^{2}}(a+c)^{HKd}\,da\,dc \int_{\eta_{2} a}^{T}\frac{1}{b ^{HKd}}\,db \\ &\leq k \int_{[0,T]^{2}}a^{-\frac{4dHK}{3}+1}c^{-\frac{2dHK}{3}}\,da\,dc< + \infty . \end{aligned}$$

For $i=3$, we also decompose the integral region as $\mathcal{T}_{3}=I _{1}+I_{2}+I_{3}+I_{4}$, where $I_{1}=\{a\geq \eta_{1}b,c\geq \eta _{2}b\}$, $I_{2}=\{a<\eta_{1}b,c<\eta_{2}b\}$, $I_{3}=\{a\geq \eta _{1}b,c<\eta_{2}b\}$, and $I_{4}=\{a<\eta_{1}b,c\geq \eta_{2}b\}$, for some fixed but arbitrary $\eta_{1}>0$ and $\eta_{2}>0$.

Firstly, we consider in the region $I_{1}$. By (3.19) of Lemma 3.3, it follows that

$$\begin{aligned} \int_{I_{1}}\varTheta_{3}\,da\,db\,dc &\leq \int_{a\geq \eta_{1}b,c\geq \eta _{2}b} k(\lambda_{i}\rho_{i})^{-\frac{d}{2}} \,da\,db\,dc \\ &=k \int_{a\geq \eta_{1}b,c\geq \eta_{2}b} \bigl(a^{2HK}c^{2HK} \bigr)^{- \frac{d}{2}}\,da\,db\,dc \\ &=k \int_{0}^{T}\,db \int_{\eta_{1}b}^{T}a^{-dKH}\,da \int_{\eta_{2}b}^{T}c ^{-dKH}\,dc \\ &\leq k \int_{0}^{T}\frac{db}{b^{2(dHK-1)}} < +\infty . \end{aligned}$$

Secondly, in the region $I_{2}$. By (3.8) and (3.18), we obtain that

$$\begin{aligned} \varTheta_{3} &\leq k\mu^{2}_{3}( \lambda_{3}\rho_{3})^{-\frac{d}{2}-1} \\ &\leq k \bigl(b^{2HK-2}ac \bigr)^{2} \bigl(a^{2HK}c^{2HK} \bigr)^{-\frac{d}{2}-1} \\ &\leq kb^{4HK-4}a^{2-2HK-dHK}c^{2-2HK-dHK} \\ &\leq ka^{-\frac{2}{3}dHK}c^{-\frac{2}{3}dHK}b^{-\frac{2}{3}dHK}, \end{aligned}$$

where we have used the inequality $-\frac{2}{3}dHK<2-2HK-dHK$. Therefore,

$$\begin{aligned} \int_{a< \eta_{1}b,c< \eta_{2}b}\varTheta_{3}\,da\,db\,dc\leq k \int_{a< \eta_{1}b,c< \eta_{2}b}a^{-\frac{2}{3}dHK}c^{-\frac{2}{3}dHK}b ^{-\frac{2}{3}dHK}\,da \,db\,dc< +\infty . \end{aligned}$$

Finally, we consider the case $a\geq \eta_{2}b$ and $c<\eta_{1}b$, the region $I_{4}$ can be achieved similarly.

If $HKd > 1$, then $2HK-1 > 0$. On the one hand, by inequality (3.9), for $\eta \in (e+a+b, e+a+b+c)$, we have

$$\begin{aligned} \Delta_{3,2}\leq kac\eta^{2HK-2} \leq kac(e+a+b)^{2HK-2} \leq kaca ^{2HK-2}=ka^{2HK-1}c. \end{aligned}$$

On the other hand,

$$\begin{aligned} \Delta_{3,1} &\leq \frac{1}{2^{K}} \bigl(\vert a+b+c \vert ^{2HK}-\vert a+b \vert ^{2HK} \bigr) \\ &=\frac{1}{2^{K}}2HK \int^{c}_{0}(a+b+x)^{2HK-1}\,dx \\ &\leq kc(a+b+c)^{2HK-1} \\ &\leq ka^{2HK-1}c, \end{aligned}$$

so,

$$\begin{aligned} \mu_{3}\leq ka^{2HK-1}c, \end{aligned}$$

it follows that

$$\begin{aligned} \varTheta_{3}\leq ka^{4HK-2}c^{2-dHK-2HK}a^{-dHK-2HK} = ka^{2HK-dHK-2}c ^{2 - dHK-2HK}. \end{aligned}$$

Therefore, we get

$$\begin{aligned} \int_{a\geq \eta_{1}b, c< \eta_{2}b} \varTheta_{3}\,da\,db\,dc &\leq \int_{a\geq \eta_{1}b, c< \eta_{2}b} a^{2HK-dHK - 2}c^{2-dHK-2HK}\,dc\,db\,da \\ &\leq k \int_{a\geq \eta_{1}b}a^{2HK-dHK - 2}b^{3-dHK-2HK}\,db\,da \\ &\leq k \int_{e\leq \eta a}a^{2HK-dHK-2}a^{4-dHK-2HK}\,da \\ &= k \int_{0}^{T} a^{2-2dHK}\,da< + \infty . \end{aligned}$$

If $HKd \leq 1$, we get that $2HK - 1 \leq 0$, then

$$ \varTheta_{3}\leq k(\lambda_{3} \rho_{3})^{-\frac{d}{2}} \leq k \bigl(a^{2HK}c ^{2HK} \bigr)^{-\frac{d}{2}}\leq ka^{-HK}c^{-HK}. $$

Thus,

$$\begin{aligned} \int_{a\geq {\eta }_{1}b,c< {\eta }_{2}b}{\varTheta }_{3}\,da\,db\,dc &\leq k \int_{a\geq {\eta }_{1}b,c< {\eta }_{2}b} a^{-HK}c^{-HK}\,dc\,db\,da \\ &\leq k \int_{[0,T]^{3}} a^{-HK}c^{-HK}\,dc\,db\,da \\ &< + \infty . \end{aligned}$$

*Step* 2. We give the proof of the necessary condition. Assume that $dHK=\frac{3}{2}$, then we claim that $\varXi_{T}=+\infty $. It suffices to show that

3.26 $$\begin{aligned} \int_{\mathcal{T}}\mu^{2}(\lambda \rho )^{-\frac{d}{2}-1}\,ds \,dt\,ds'\,dt'=+ \infty . \end{aligned}$$

In order to get this result, we just prove the result for $i=3$. Because $\mu_{3}=\Delta_{3,1}+\Delta_{3,2}$, then

3.27 $$\begin{aligned} & \int_{\mathcal{T}}\mu_{3}^{2}(\lambda_{3} \rho_{3})^{-\frac{d}{2}-1}\,ds\,dt\,ds'\,dt' \\ &\quad = \int_{\mathcal{T}}\Delta_{3,1}^{2}( \lambda_{3}\rho_{3})^{-\frac{d}{2}-1}\,ds\,dt \,ds'\,dt' + \int_{\mathcal{T}}\Delta_{3,2}^{2}(\lambda _{3}\rho_{3})^{-\frac{d}{2}-1}\,ds\,dt\,ds' \,dt' \\ & \qquad {}+2 \int_{\mathcal{T}}\Delta_{3,1}\Delta_{3,2}( \lambda_{3}\rho_{3})^{- \frac{d}{2}-1}\,ds\,dt \,ds'\,dt' \\ &\quad :=A_{1}+A_{2}+A_{3}, \end{aligned}$$

where

$$ A_{1}:= \int_{\mathcal{T}}\Delta_{3,1}^{2}( \lambda_{3}\rho_{3})^{- \frac{d}{2}-1}\,ds\,dt \,ds'\,dt', \qquad A_{2}:= \int_{\mathcal{T}}\Delta_{3,2} ^{2}( \lambda_{3}\rho_{3})^{-\frac{d}{2}-1}\,ds\,dt \,ds'\,dt', $$

and

$$ A_{3}:=2 \int_{\mathcal{T}}\Delta_{3,1}\Delta_{3,2}( \lambda_{3}\rho _{3})^{-\frac{d}{2}-1}\,ds\,dt \,ds'\,dt'. $$

When $2HK-1>0$, for the term $A_{1}$, by Lemma 11 of Hu [7], it is easy to get

3.28 $$\begin{aligned} A_{1}=+\infty . \end{aligned}$$

Now, we consider the term $A_{2}$. Because $2HK-1>0$, we have $2H-1>0$, $d=2$, and $HK=\frac{3}{4}$. It is easy to get from (3.9) that there exists a constant $k>0$ such that

3.29 $$\begin{aligned} \Delta_{3,2} &\geq k\xi^{2H-1} \eta^{2H-1} \bigl[\xi^{2H}+\eta^{2H} \bigr]^{K-2}ac \\ &\geq k\xi^{2H-1}\eta^{2HK-2H-1}ac \\ &\geq ke^{2H-1}(a+b+c+e)^{2HK-2H-1}ac. \end{aligned}$$

Then we have that, for $a,b,c,e>0$,

3.30 $$\begin{aligned} A_{2} &= \int_{0< a+b+c+e< T}\Delta_{3,2}^{2}( \lambda_{3}\rho_{3})^{- \frac{d}{2}-1}\,da\,db\,dc\,de \\ &\geq k \int_{[0,\varepsilon ]^{4}}e^{4H-2}(a+b+c+e)^{2(2HK-2H-1)}(ac)^{2-HKd-2HK} \,da\,db\,dc\,de \\ &\geq k \int_{[0,\varepsilon ]^{4}}e^{4H-2}(ac)^{-1}\,da\,db\,dc\,de \\ &= +\infty . \end{aligned}$$

Next, for the term $A_{3}$, following the inequalities

3.31 $$\begin{aligned} \Delta_{3,1} &=\frac{1}{2^{K}} \bigl[(a+b+c)^{2HK}-(a+b)^{2HK}-(b+c)^{2HK}+(b)^{2HK} \bigr] \\ &=\frac{1}{2^{K}}\cdot 2HK\cdot (2HK-1)ac \int_{0}^{1} \int_{0}^{1}(b+va+ua)^{2HK-2}\,du\,dv \\ &\geq k(a+b+c)^{2HK-2}ac \end{aligned}$$

and (3.29), we get

$$\begin{aligned} \frac{2\Delta_{3,1}\Delta_{3,2}}{(\lambda \rho )^{\frac{d}{2}+1}} & \geq k(a+b+c)^{2HK-2}d^{2H-1}(a+b+c+e)^{2HK-2H-1}(ac)^{2-2HK-HKd} \\ &= k(a+b+c)^{-\frac{1}{2}}e^{2H-1}(a+b+c+e)^{\frac{1}{2}-2H}(ac)^{-1} \geq ka^{-1}c^{-1}e^{2H-1}. \end{aligned}$$

So,

3.32 $$\begin{aligned} \frac{A_{3}}{2}&= \int_{0< a+b+c+e< T}\Delta_{3,1}\Delta_{3,2}(\lambda _{3}\rho_{3})^{-\frac{d}{2}-1}\,da\,db\,dc\,de \\ &\geq \int_{[0,\varepsilon ]}a^{-1}c^{-1}e^{2H-1}\,da \,db\,dc\,de= +\infty . \end{aligned}$$

By combining inequalities (3.28), (3.30) with (3.32), we get that $\varXi_{T}=+\infty $.

When $2HK-1\leq 0$, we have $d\geq 3$ and $2-HKd-2HK=\frac{1}{2}-\frac{3}{d}>-1$. In order to check (3.26), we use a similar way of the proof of Lemma 11 in Hu [7]. For convenience, we give shortly the proof. Notice that

$$\begin{aligned} \mu_{3}^{2}=(\Delta_{3,1}+\Delta_{3,2})^{2} \geq \Delta_{3,1}^{2} \geq k(a+b+c)^{4HK-4}a^{2}c^{2}. \end{aligned}$$

It follows that

$$\begin{aligned}& \int_{\mathcal{T}}\mu_{3}^{2}(\lambda_{3} \rho_{3})^{-\frac{d}{2}-1}\,ds\,dt\,ds'\,dt' \\& \quad \geq k \int_{\mathcal{T}}(a+b+c)^{4HK-4}a^{2}c^{2} \bigl((ac)^{2HK} \bigr)^{- \frac{d}{2}-1}\,da\,db\,dc \\& \quad \geq k \int_{[0,\varepsilon ]^{3}}(a+b+c)^{4HK-4}(ac)^{2-HKd-2HK}\,da\,db\,dc \\& \quad =\frac{k}{3-4HK} \int_{[0,\varepsilon ]^{2}} \bigl[(a+c)^{4HK-3}-(a+c+ \varepsilon )^{4HK-3} \bigr](ac)^{2-HKd-2HK}\,da\,dc \end{aligned}$$

and

$$\begin{aligned} \int_{0< a< c< \varepsilon }(a+c)^{4HK-3}(ac)^{2-HKd-2HK}\,da\,dc& \geq k \int_{0< a< c< \varepsilon }a^{2-HKd-2HK}c^{2HK-HKd-1}\,da\,dc \\ &\geq k \int_{0}^{\varepsilon }a^{2-2HKd}\,da = +\infty , \end{aligned}$$

where $2HK-HKd-1<-1$. This completes the proof of this lemma. □

Now, we give the proof of Theorem 1.2.

### Proof of Theorem 1.2

By (3.4), we have

3.33 $$\begin{aligned} &\lim_{\varepsilon_{1}\rightarrow 0, \varepsilon_{2}\rightarrow 0} \mathbb{E} \bigl(L_{\varepsilon_{1}}(H,K,T)-\mathbb{E} \bigl[L_{\varepsilon _{1}}(H,K,T) \bigr] \bigr) \bigl(L_{\varepsilon_{2}}(H,K,T)- \mathbb{E} \bigl[L_{ \varepsilon_{2}}(H,K,T) \bigr] \bigr) \\ &\quad =\varXi_{T}. \end{aligned}$$

And it follows from Lemma 3.4 that

$$\begin{aligned} \varXi_{T}= \int_{\mathcal{T}} \bigl[ \bigl(\lambda \rho -\mu^{2} \bigr)^{-\frac{d}{2}}-( \lambda \rho )^{-\frac{d}{2}} \bigr]\,d\tau < +\infty\quad \mbox{if and only if } HKd< \frac{3}{2}. \end{aligned}$$

These imply that the renormalized self-intersection local time $L_{\varepsilon }(H,K,T)-\mathbb{E}[L_{\varepsilon }(H,K,T)]$ of $B^{H,K}$ converges in $L^{2}$ as *ε* tends to zero if and only if $HKd<\frac{3}{2}$. This completes the proof of Theorem 1.2. □

## Conclusions

In this paper, we considered that the local time and the renormalized self-intersection local time of *d*-dimensional bifractional Brownian motion with Hurst parameters $H\in (0,1)$ and $K\in (0,1]$ exist in $L^{2}$ for $d\geq 2$. Our work generalizes the results of the local time of fractional Brownian motion in Hu and Øksendal [8] and the renormalized self-intersection local time of fractional Brownian motion in Hu and Nualart [7], respectively.
